# Gene coexpression network approach to develop an immune prognostic model for pancreatic adenocarcinoma

**DOI:** 10.1186/s12957-021-02201-w

**Published:** 2021-04-12

**Authors:** Xiaoqiang Gu, Qiqi Zhang, Xueying Wu, Yue Fan, Jianxin Qian

**Affiliations:** 1 Department of Oncology, Longhua Hospital Affiliated to Shanghai University of Traditional Chinese Medicine, Shanghai, China; 2 Department of Integrated traditional Chinese and Western Medicine, Zhongshan Hospital of Fudan University, Shanghai, China; 3 Fudan Zhongshan Cancer Center, Zhongshan Hospital of Fudan University, Shanghai, China; 4 Genecast Biotechnology Co., Ltd., 88 Danshan Road, Xidong Chuangrong Building, Suite D-401, Wuxi City, Jiangsu China

**Keywords:** Pancreatic cancer, WGCNA, Immune prognostic model, Immune profile, Immunotherapy

## Abstract

**Background:**

Pancreatic adenocarcinoma (PAAD) is a nonimmunogenic tumor, and very little is known about the relationship between the host immune response and patient survival. We aimed to develop an immune prognostic model (IPM) and analyze its relevance to the tumor immune profiles of patients with PAAD.

**Methods:**

We investigated differentially expressed genes between tumor and normal tissues in the TCGA PAAD cohort. Immune-related genes were screened from highly variably expressed genes with weighted gene correlation network analysis (WGCNA) to construct an IPM. Then, the influence of IPM on the PAAD immune profile was comprehensively analyzed.

**Results:**

A total of 4902 genes highly variably expressed among primary tumors were used to construct a weighted gene coexpression network. One hundred seventy-five hub genes in the immune-related module were used for machine learning. Then, we established an IPM with four core genes (*FCGR2B*, *IL10RA*, and *HLA-DRA*) to evaluate the prognosis. The risk score predicted by IPM was an independent prognostic factor and had a high predictive value for the prognosis of patients with PAAD. Moreover, we found that the patients in the low-risk group had higher cytolytic activity and lower innate anti-PD-1 resistance (IPRES) signatures than patients in the high-risk group.

**Conclusions:**

Unlike the traditional methods that use immune-related genes listed in public databases to screen prognostic genes, we constructed an IPM through WGCNA to predict the prognosis of PAAD patients. In addition, an IPM prediction of low risk indicated enhanced immune activity and a decreased anti-PD-1 therapeutic response.

**Supplementary Information:**

The online version contains supplementary material available at 10.1186/s12957-021-02201-w.

## Introduction

Pancreatic adenocarcinoma (PAAD) is historically one of the deadliest malignancies; the 5-year survival rate is less than 9% for patients with all stages combined [1]. Furthermore, the incidence of PAAD is rapidly increasing worldwide and is predicted to become the second leading cause of cancer-related death in the future, behind only lung cancer [2]. Treatment options for advanced PAAD are limited to gemcitabine-based chemotherapy and are hampered by ineffective clinical outcomes.

Immune surveillance is the first filter to identify and eliminate aberrant or malignant cells [3], and PAAD is regarded as a nonimmunogenic tumor [4]. Hence, most tumor cells avoid recognition by host immune cells, promoting PAAD progression. Although several immune-related parameters, such as CD8^+^ T cell infiltration and PD-L1 expression, have been reported to predict the prognosis of patients with PAAD [5], immune prognostic models (IPMs) have not yet been developed for PAAD.

Weighted gene correlation network analysis (WGCNA) is a powerful approach in network modeling that is useful for identifying cluster modules of highly related genes, summarizing these clusters with module eigengenes (MEs) or intramodular hub genes, and relating modules to one another and to external sample traits [6]. Currently, WGCNA has been widely used in various cancer-related studies to identify candidate biomarkers [7, 8].

In the present study, we first built the WGCNA network using primary tumors from 177 patients in The Cancer Genome Atlas (TCGA) database. The WGCNA network was constructed by highly variably expressed among tumors, and immune scores calculated using the “ESTIMATE” package [9] were used to identify immune-related modules. By selecting hub genes in these immune-related modules, this study developed an IPM that may identify low-risk patients. Importantly, our investigation indicated that low-risk patients predicted by the IPM present higher immune activity and might experience a greater benefit from immunotherapies than high-risk patients.

## Materials and methods

### Data collection

The RNA high-throughput sequencing (RNA-HTSeq) read count data and clinical information from patients with PAAD (*n*=177) were obtained from the GDC Data Portal (https://portal.gdc.cancer.gov/). Normal tissue (*n*=171) expression was obtained from the GTEx portal (https://www.gtexportal.org/).

### Pre-processing

The following pre-processing method was used: (1) removing genes with counts ≤ 1 in all samples and (2) normalizing expression data by variance stabilizing transformation (VST). Highly variably expressed genes (HVGS) were analyzed in R (version: 3.6.3), and a cutoff of top quartile (>25%) are considered hypervariable.

### WGCNA

The gene coexpression networks were constructed using the WGCNA package [6]. A coexpression network was constructed for all differentially expressed genes, and the Pearson correlation coefficients were calculated between all genes. A β (soft-thresholding power) parameter was determined based on the scale-free topology of the network to reconstruct the network with strongly correlated genes and eliminate weakly correlated genes.

### Evaluation of the levels of immune cell infiltration and tumor purity

The levels of immune cell infiltration (immune score) and tumor purity for each PAAD sample were calculated using the “estimateScore” function in the “ESTIMATE” package [9]. This method is based on the single sample gene set enrichment analysis (ssGSEA) algorithm [10].

### Construction of an immune-related prognostic model

The expression profiles of 2991 genes from patients in TCGA PAAD cohort with survival information were analyzed using a univariate Cox regression analysis. Ten variables with both a log-rank *P*≤0.2 and likelihood *P*≤0.2 in the univariate analysis were entered into the primary IPM. Next, the “step” function in the survival “package” with the option direction “both” compares improvements in the Akaike information criterion (AIC) achieved by systematically deleting each candidate variable and adding each candidate variable between the upper and lower bound regressor sets supplied from the current model and by deleting or adding the one variable with best improvement in the AIC (smallest AIC).

### Analysis of the immune-related signature

Gene sets for cytolytic activity (granzyme-A, perforin-1) [11] and activated CD8 T cells [12] were used in previous studies. TCGA transcriptomic profiling data were obtained from the GDC Data Portal (https://portal.gdc.cancer.gov/). The expression of each target gene was normalized by TPM, and the immune signatures were measured as the geometric mean of gene expression reported as the log2 value of TPM+1.

The innate anti-PD-1 resistance (IPRES) signatures were calculated based on the average *Z* score across all gene sets associated with tumor metastasis, as described in a published study [13].

### The association between the risk score and survival outcome

Kaplan-Meier survival curves were used to show differences in survival, and the log-rank test was used to evaluate the significance of differences in survival times. We performed survival analyses using the R programming function “survfit” in the “survival” package.

### Statistical analysis

All statistical analyses were performed using R version 3.6.3 software (Institute for Statistics and Mathematics, Vienna, Austria; www.r-project.org). The Wilcoxon test was used to compare two continuous variables. Survival was analyzed by constructing a Kaplan-Meier survival plot, and the log-rank test calculated the *P* value. *P* < 0.05 was considered statistically significant.

## Results

### Construction of the WGCNA

**Fig. 1:**
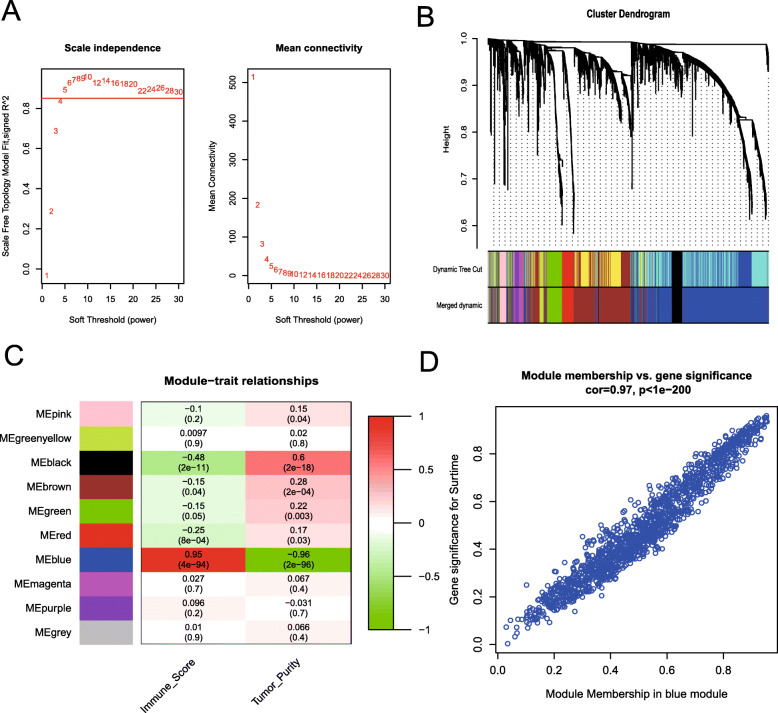
Construction of the gene coexpression network for PAAD. **a** Checking the scale-free topology when *β* = 12. **b** The consensus gene dendrogram and corresponding module colors are shown. The vertical axis represents the gene expression value, and the horizontal axis represents the genes. Each vertical line in the dendrogram relates to a gene, and each branch indicates highly coexpressed genes as a module (one color). Twelve modules were detected and merged into 10 main modules. **c** Module-trait relationships. Each row represents a ME, the two columns represent the immune score and tumor purity, and each cell contains the corresponding correlation and *P* value. The matrix is color-coded by correlation according to the color legend. **d** Scatterplot of gene significance (*y*-axis) vs. module membership (*x*-axis) in the most significant module (pink module, see panel **c**)

The coexpression network was constructed using the expression of 4902 HVGs in 177 PAAD samples. For building signed networks with WGCNA, we choose *β*=12 to compute gene co-expression similarity, resulting in a scale-free topology index (*R*^2^) of 0.93 (Fig. 1a). Eighteen coexpression modules were constructed and are shown in different colors (Fig. 1b). MEs were examined and the dynamic modules were merged into 11 modules with a threshold of 0.25 to increase the reliability of the module divisions (Fig. 1b).

### Identification of module-trait relationships and hub genes

We further wanted to determine which module is more closely related to immune profiles after identifying the MEs. As described in a previous study, immune estimation approaches should be tested to determine whether they display a negative correlation with tumor purity [14]. Hence, we calculated the immune score and tumor purity for each PAAD sample to screen immune-related modules (see the “Materials and methods” section). Figure 1c shows the module-trait heat map. The pink module exhibited the highest correlation with the immune score (*R* = 0.98, *P* < 0.001) and a significant negative correlation with tumor purity (*R* = −0.95, *P* < 0.001). Therefore, the pink module containing 640 genes was identified as the module with immune significance.

Hub genes are highly interconnected with nodes in a module and have been shown to be functionally significant. In the present study, hub genes were defined by module connectivity, as measured by the absolute value of Pearson’s correlation coefficient (module membership (MM) > 0.8), and by trait relationship, which was measured by the absolute value of Pearson’s correlation coefficient (gene significance (GS) > 0.8). Eventually, 175 highly connective genes in the pink module were selected as hub genes (Table S1). Moreover, GS and MM were highly correlated in the pink module (Fig. 1d), implying that hub genes of the pink module also tend to be highly correlated with immune profiles.

### Functional enrichment analysis of hub genes in the pink module

**Fig. 2:**
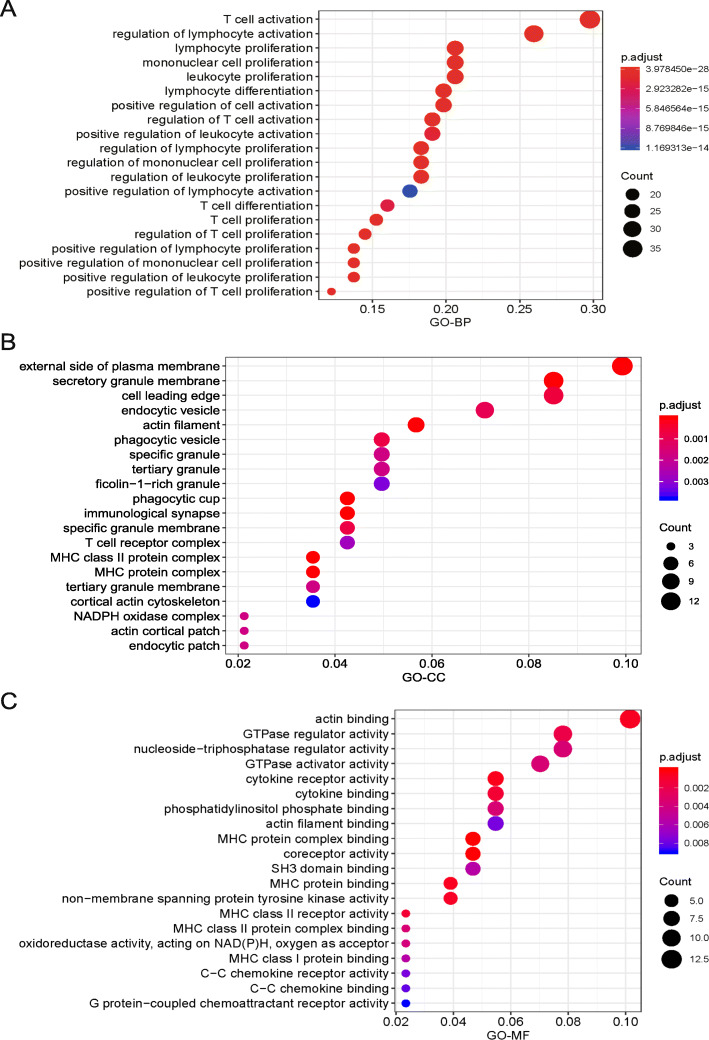
Analysis of enriched GO terms for the hub genes in the pink module. The analysis of enriched GO terms was performed using the function “enrichGO” in the “clusterProfiler” package. Biological process (BP, panel **a**); cellular component (CC, panel **b**); molecular function (MF, panel **c**). The *y*-axis represents the number of genes associated with the GO term. The intensity and color of dots are indicated on the right side of the heatmap and are represented by their corresponding adjusted *P* values

Before developing an IPM for pancreatic cancer, we must verify whether the hub genes are related to immunity. Therefore, we performed an enrichment analysis of Gene Ontology (GO) terms associated with the hub genes in the pink module using the R programming function “enrichGO” in the “clusterProfiler” package [15]. The top 20 clustering groups obtained in each root category (biological process (BP), cellular component (CC), and molecular function (MF)) in the GO enrichment analysis are shown separately in Fig. 2. Hub genes in the pink module were highly enriched in pathways closely related to regulation of lymphocyte activation (Fig. 2a), MHC protein complexes (Fig. 2b), and cytokine receptor activity (Fig. 2c). Taken together, we identified an immune-related gene set in PAAD based on WGCNA.

### Construction of an IPM and evaluation of its predictive ability

The univariate Cox regression analysis revealed that 10 of these hub genes in the pink module were probably related to the overall survival (OS) of patients with PAAD (Table S2). A primary IPM was constructed by performing a multivariate Cox regression analysis of the expression levels of 10 immune-related genes. The optimized IPM was chosen by determining the maximum AIC in a stepwise regression analysis in both directions using the R package (see the “Materials and methods” section). Three core genes (*FCGR2B*, *IL10RA*, and *HLA-DRA*) were included in the optimized IPM, and the covariates *IL10RA* (hazard ratio (HR) 0.36; 95% CI 0.24−0.52, *P* < 0.001), *FCGR2B* (HR 1.46; 95% CI 1.03−2.06, *P* = 0.033), and *HLA-DRA* (HR 1.61; 95% CI 1.12−2.32, *P* = 0.01) were significant (Figure S1A). The risk score for each sample was calculated utilizing the regression coefficients derived from the multivariate Cox regression analysis to multiply the normalized expression level of the 3 core genes. As shown in Figure S1B-D, the expression levels of *FCGR2B*, *IL10RA*, and *HLA-DRA* in tumor samples (*n*=177) were significantly higher (*q* value < 0.001; log2FoldChange > 1) than in normal pancreatic tissue (*n*=171; from *GTEx* database).

**Fig. 3:**
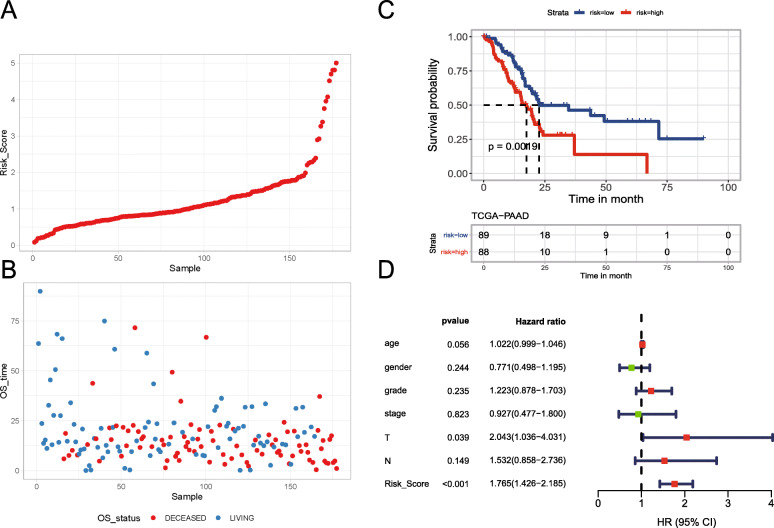
Analysis of the prognostic value of the IPM. The risk scores (**a**) and OS (**b**) of each patient. The patients were ranked by risk score. The dot plot shows the survival status of each patient. Red: deceased; pink: alive. Kaplan-Meier survival curves showing the OS times of patients stratified into low/high-risk groups (**c**). *P* values were obtained from the log-rank test. Forest plot of the multivariate Cox regression proportional hazards regression analysis of OS in TCGA PAAD cohort (**d**). CI, confidence interval; HR, hazard ratio

As risk scores increased, the patient mortality risk increased and the OS decreased (Fig. 3a, b). The cut-off point (0.96) was set as the median risk score to assign patients into high- and low-risk groups. As shown in Fig. 3c, low-risk patients with PAAD (*n*=89) had a significantly longer OS than high-risk patients with PAAD (*n*=88) (log-rank test, *P* < 0.001). Furthermore, the risk score was independently significant and the most valuable prognostic factor in TCGA PAAD cohort (HR 1.6; 95% CI 1.33–2.0, *P* < 0.001; Fig. 3d).

### A low risk score indicated increased immune activity and reduced features of the response to anti-PD-1 therapy

**Fig. 4:**
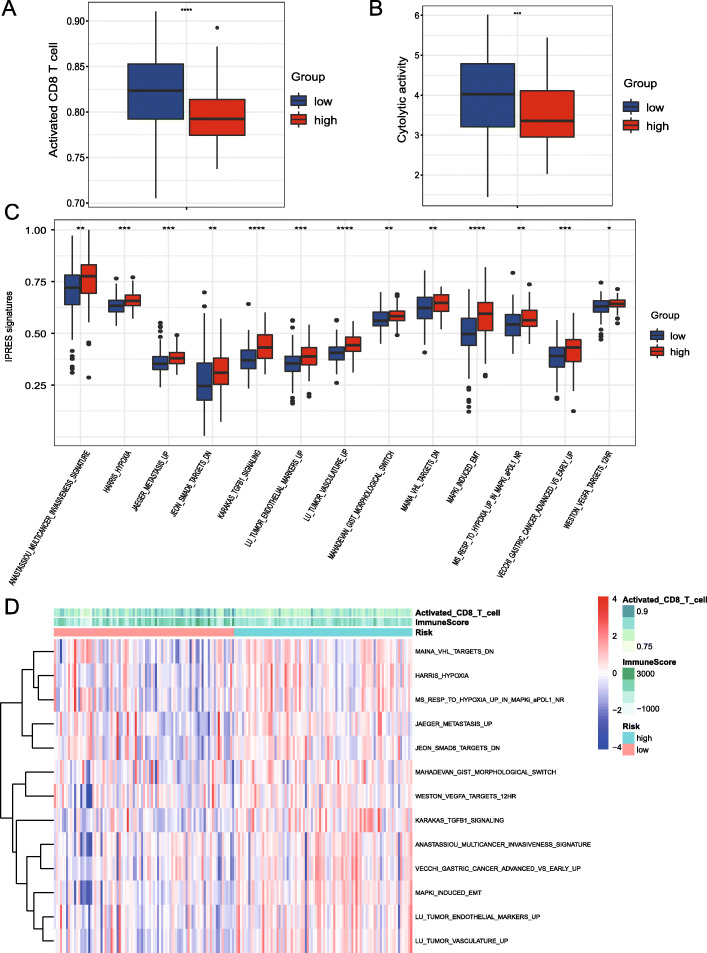
Associations between low/high risks and immune profiles. The activated CD8^+^ T cell fraction (**a**) and cytolytic activity (**b**) in the low-risk PAAD group were significantly increased compared with the high-risk group. The IPRES signatures of the low-risk PAAD group were significantly decreased compared with the high-risk group (**c**). *P* values were calculated with the Wilcoxon test; the box shows the upper and lower quartiles (*P < 0.05, **P < 0.01, and ***P < 0.001). Heatmap showing scores for IPRES signatures in TCGA PAAD cohort (**d**)

Since the prognostic model was constructed based on immune-related genes, we wanted to further explore whether patients with different risk scores have different immune profiles and subsequently differences in sensitivity to immunotherapies. The status of tumor-infiltrating lymphocytes (TILs), particularly CD8^+^ T cells, has been verified as the core determinant of immune checkpoint inhibitor (ICI) treatment efficacy [16, 17]. Low-risk patients with PAAD presented significantly greater numbers of activated CD8^+^ T cells than high-risk patients with PAAD (Wilcoxon test, *P* < 0.001; Fig. 4a). Additionally, cytolytic T cell activity (CYT value), which predicts the immunotherapy response [11, 18], was also higher in low-risk patients with PAAD than in high-risk patients with PAAD (Wilcoxon test, *P* < 0.001; Fig. 4b).

On the other hand, an attenuation of the biological processes that underlie IPRES signatures may improve the response of various cancer types to anti-PD-1 therapy, including PAAD [13]. Interestingly, as shown in Fig. 4 c, d , multiple IPRES signatures, including the mesenchymal transition, angiogenesis, and hypoxia, were significantly downregulated in the low-risk group. Collectively, a low-risk status is associated with increased immune activity and the IPM prediction of a low-risk status may serve as an indicator of the response of PAAD to ICIs.

## Discussion

Previous studies have noted the strong relationship between the host immune response and survival of patients with cancer [19–21]. Researchers frequently focus on using well-established immune-related gene sets to screen prognostic genes. However, a number of fundamental questions and problems remain to be solved; one of these problems is that this method ignores differences in the cancer types. Compared with other cancers, PAAD has unique immunological conditions with a dense stromal environment and a highly immunosuppressive tumor microenvironment [22], implying the potential for specific transcriptomic features of pancreatic cancer. Therefore, researchers have not been able to easily determine whether the included immune-related gene set accurately represents the characteristics of PAAD. The WGCNA efficiently identifies modular immune-related genes in specific cancer types to address this problem. This technology focuses on the relationship between gene modules and disease traits instead of linking thousands of genes to the disease. Consequently, hidden key genes associated with a particular biological process in cancer can be discovered using WGCNA.

In the present study, we constructed a prognostic model for PAAD using four immune-related genes (*FCGR2B*, *IL10RA*, and *HLA-DRA*) identified by WGCNA and machine learning. To the best of our knowledge, the genes that constitute our IPM have not yet been reported to be associated with the prognosis of patients with PAAD. *FCGR2B*, an inhibitory Fcgamma receptor [23], has been proven to be a prognostic factor for patients with glioblastoma [24]. The prognostic values of *IL10RA* and *HLA-DRA* have been considered even lower in the literature*.* However, in the multivariate Cox analysis, the covariates *IL10RA* and *HLA-DRA* remained significant in PAAD. Additionally, patients classified into the high-risk group had significantly worse outcomes, and the ROC analysis verified that the risk score had good prognostic accuracy. Based on these results, the IPM was successfully established to assess the prognosis of patients with PAAD.

In the era of immunotherapy, pancreatic cancer remains an incurable disease because of an immunosuppressive and anti-inflammatory environment that results in the escape of cancer cells from such therapies [25]. Nevertheless, some advances have been achieved using therapy targeting PD-1 and its ligand PD-L1 [26, 27], suggesting that some patients with PAAD indeed benefit from ICIs. To date, the microsatellite instability (MSI) or mismatch repair deficiency (dMMR) status remains the only clear marker of a benefit from PD-1 blockade in patients with PAAD. Unfortunately, probably less than 1% of patients with PAAD have a high MSI (MSI-H) or dMMR [28], implying a pressing need to identify potential predictive therapeutic biomarkers that will help patients derive greater benefits from ICIs. Perhaps, the most unexpected finding in our study is that low-risk PAAD is associated with increased cytolytic cell infiltration, indicating an inflammatory phenotype. This type of PAAD suggests the presence of a pre-existing anticancer immune response [29], and the majority of patients with inflamed tumors will show an evident clinical response to anti-PD-1/PD-L1 monotherapy [30]. However, the immune infiltrate of pancreatic cancer is a part of the tumor microenvironment. IPRES, a transcriptomic signature associated with increased neoplasm invasion and angiogenesis, is associated with resistance to PD-1 blockade [13]. PAAD is a cancer type that contains IPRES-enriched transcriptomic subsets involved in the majority of tumors [13]. Interestingly, based on our data, the IPRES signatures in low-risk patients with PAAD were significantly reduced compared to high-risk patients. Thus, low-risk patients may benefit from ICIs.

This study has several limitations. First, an external validation cohort is needed to verify the validity of the IPM prediction. Second, without data from an ICI-treated cohort, we were unable to evaluate whether patients with low-risk PAAD exhibit a better response to ICIs than high-risk patients. Therefore, the study findings should be interpreted with caution, and further studies are warranted.

## Conclusions

In summary, we identified and validated an IPM based on 3 immune-related genes with independent prognostic significance for patients with PAAD. In addition, the present study proposed that the risk score may be an immunotherapeutic marker for PAAD.

## Supplementary Information

**Additional file 1: Figure S1**. Evaluation of predicted outcomes. Forest plot of the multivariate Cox regression proportional hazards regression analysis of OS in TCGA PAAD cohort (A). *FCGR2B* (B), *IL10RA* (C), and *HLA-DRA* (B) expression between tumor and normal tissue.

**Additional file 2: Table S1**. Hub genes in the pink module.

**Additional file 3: Table S2**. Ten genes with both a log-rank *P* ≤ 0.2 and likelihood *P* ≤ 0.2 in the univariate analysis.

## Data Availability

All data used in the manuscript were extracted from the public database indicated in the manuscript.

## Abbreviations

PAAD: Pancreatic adenocarcinoma
IPM: Immune prognostic model
IPRES: Innate anti-PD-1 resistance
WGCNA: Weighted gene correlation network analysis
MEs: Module eigengenes
TCGA: The Cancer Genome Atlas
AIC: Akaike information criterion
GO: Gene Ontology
BP: Biological process
CC: Cellular component
MF: Molecular function
ROC: Receiver operating characteristic
TILs: Tumor-infiltrating lymphocytes
ICI: Immune checkpoint inhibitor
MSI: Microsatellite instability
dMMR: Mismatch repair deficiency
