# Supplementary material for: Gene coexpression network approach to develop an immune prognostic model for pancreatic adenocarcinoma
Source: World J Surg Oncol. 2021 Apr 12;19:112. doi: 10.1186/s12957-021-02201-w (PMC8042890; doi:10.1186/s12957-021-02201-w)
Supplement: Supplementary file 2 — Additional file 2: Table S1. Hub genes in the pink module. [file 12957_2021_2201_MOESM2_ESM.docx]

**Table S1**

**Hub genes in the pink module.**

| geneSymbol | moduleColor | GS.Immune.Score | p.GS.Immune.Score | MM.pink | p.MM.pink |
| --- | --- | --- | --- | --- | --- |
| SASH3 | pink | 0.951092 | 3.32E-91 | 0.97337 | 6.95E-114 |
| BTK | pink | 0.951172 | 2.89E-91 | 0.97312 | 1.56E-113 |
| CD53 | pink | 0.959465 | 3.51E-98 | 0.969746 | 4.19E-109 |
| WAS | pink | 0.951556 | 1.47E-91 | 0.964832 | 1.77E-103 |
| HCLS1 | pink | 0.934623 | 1.72E-80 | 0.964441 | 4.59E-103 |
| IKZF1 | pink | 0.919552 | 6.74E-73 | 0.960522 | 3.63E-99 |
| NCKAP1L | pink | 0.937348 | 4.68E-82 | 0.96044 | 4.35E-99 |
| WDFY4 | pink | 0.92136 | 9.99E-74 | 0.959598 | 2.64E-98 |
| DOCK2 | pink | 0.933906 | 4.33E-80 | 0.959107 | 7.45E-98 |
| BIN2 | pink | 0.948791 | 1.68E-89 | 0.951573 | 1.43E-91 |
| FAM78A | pink | 0.916116 | 2.24E-71 | 0.947158 | 2.44E-88 |
| ITGAL | pink | 0.933774 | 5.12E-80 | 0.946687 | 5.18E-88 |
| EVI2B | pink | 0.943449 | 7.82E-86 | 0.943142 | 1.24E-85 |
| ARHGAP30 | pink | 0.925563 | 9.84E-76 | 0.941837 | 8.53E-85 |
| PLEK | pink | 0.930374 | 3.52E-78 | 0.9407 | 4.41E-84 |
| RCSD1 | pink | 0.88526 | 4.49E-60 | 0.937296 | 5.02E-82 |
| PTPRC | pink | 0.906765 | 1.53E-67 | 0.93728 | 5.13E-82 |
| IL10RA | pink | 0.907597 | 7.27E-68 | 0.934649 | 1.66E-80 |
| PIK3R5 | pink | 0.912243 | 9.80E-70 | 0.934427 | 2.22E-80 |
| ARHGAP25 | pink | 0.897157 | 5.32E-64 | 0.932346 | 3.11E-79 |
| KLHL6 | pink | 0.913271 | 3.66E-70 | 0.932299 | 3.30E-79 |
| CD48 | pink | 0.916865 | 1.06E-71 | 0.932235 | 3.57E-79 |
| TAGAP | pink | 0.906792 | 1.50E-67 | 0.932132 | 4.06E-79 |
| TRAF3IP3 | pink | 0.904474 | 1.16E-66 | 0.93118 | 1.32E-78 |
| SLA | pink | 0.916333 | 1.81E-71 | 0.930288 | 3.91E-78 |
| SCIMP | pink | 0.896366 | 1.00E-63 | 0.929218 | 1.41E-77 |
| LCP2 | pink | 0.918484 | 2.04E-72 | 0.929114 | 1.60E-77 |
| CD37 | pink | 0.901562 | 1.41E-65 | 0.92508 | 1.70E-75 |
| CD180 | pink | 0.908476 | 3.28E-68 | 0.923826 | 6.86E-75 |
| ARHGAP9 | pink | 0.907992 | 5.09E-68 | 0.923592 | 8.88E-75 |
| TNFSF8 | pink | 0.887306 | 1.02E-60 | 0.92332 | 1.20E-74 |
| GPR65 | pink | 0.914383 | 1.24E-70 | 0.923049 | 1.61E-74 |
| SNX20 | pink | 0.930346 | 3.64E-78 | 0.922599 | 2.63E-74 |
| FLI1 | pink | 0.864046 | 4.78E-54 | 0.922277 | 3.73E-74 |
| TIGIT | pink | 0.91217 | 1.05E-69 | 0.920914 | 1.61E-73 |
| CD3E | pink | 0.907337 | 9.19E-68 | 0.919843 | 4.97E-73 |
| CXorf21 | pink | 0.895974 | 1.37E-63 | 0.919746 | 5.50E-73 |
| ARHGAP15 | pink | 0.875619 | 3.37E-57 | 0.918549 | 1.91E-72 |
| PARVG | pink | 0.905556 | 4.49E-67 | 0.918063 | 3.14E-72 |
| CD2 | pink | 0.915728 | 3.30E-71 | 0.91766 | 4.73E-72 |
| CCR5 | pink | 0.904171 | 1.51E-66 | 0.914923 | 7.32E-71 |
| SLAMF1 | pink | 0.901861 | 1.09E-65 | 0.914728 | 8.87E-71 |
| CSF2RB | pink | 0.88645 | 1.90E-60 | 0.913405 | 3.22E-70 |
| MAP4K1 | pink | 0.877619 | 8.94E-58 | 0.910938 | 3.37E-69 |
| GIMAP4 | pink | 0.856824 | 3.18E-52 | 0.910428 | 5.42E-69 |
| LILRB1 | pink | 0.89244 | 2.17E-62 | 0.909427 | 1.37E-68 |
| CD3G | pink | 0.892604 | 1.92E-62 | 0.908265 | 3.97E-68 |
| SIRPG | pink | 0.897523 | 3.96E-64 | 0.908158 | 4.37E-68 |
| EVI2A | pink | 0.905275 | 5.75E-67 | 0.907834 | 5.87E-68 |
| SH2D1A | pink | 0.882657 | 2.84E-59 | 0.906497 | 1.95E-67 |
| CD28 | pink | 0.866035 | 1.44E-54 | 0.903985 | 1.77E-66 |
| IL16 | pink | 0.859742 | 6.00E-53 | 0.903498 | 2.70E-66 |
| CD4 | pink | 0.89835 | 2.02E-64 | 0.903425 | 2.87E-66 |
| TESPA1 | pink | 0.866203 | 1.30E-54 | 0.903117 | 3.74E-66 |
| SPN | pink | 0.88613 | 2.40E-60 | 0.89925 | 9.66E-65 |
| GAPT | pink | 0.867215 | 7.01E-55 | 0.898428 | 1.90E-64 |
| MYO1F | pink | 0.902362 | 7.14E-66 | 0.898394 | 1.95E-64 |
| ITK | pink | 0.867405 | 6.24E-55 | 0.898261 | 2.17E-64 |
| CD96 | pink | 0.889942 | 1.45E-61 | 0.89744 | 4.23E-64 |
| MPEG1 | pink | 0.852498 | 3.52E-51 | 0.897186 | 5.20E-64 |
| ARHGEF6 | pink | 0.838226 | 5.89E-48 | 0.896937 | 6.35E-64 |
| RNASE6 | pink | 0.881162 | 8.03E-59 | 0.89598 | 1.36E-63 |
| PYHIN1 | pink | 0.865312 | 2.23E-54 | 0.894855 | 3.32E-63 |
| RASAL3 | pink | 0.869497 | 1.71E-55 | 0.894491 | 4.43E-63 |
| GMFG | pink | 0.886641 | 1.66E-60 | 0.893239 | 1.17E-62 |
| PTPN7 | pink | 0.887548 | 8.54E-61 | 0.891891 | 3.31E-62 |
| CR1 | pink | 0.862191 | 1.44E-53 | 0.891515 | 4.41E-62 |
| SIT1 | pink | 0.865585 | 1.89E-54 | 0.891325 | 5.10E-62 |
| C1orf162 | pink | 0.876884 | 1.46E-57 | 0.890949 | 6.78E-62 |
| LY86 | pink | 0.891136 | 5.89E-62 | 0.888451 | 4.40E-61 |
| ICOS | pink | 0.883944 | 1.15E-59 | 0.887095 | 1.19E-60 |
| RASSF2 | pink | 0.84005 | 2.38E-48 | 0.886836 | 1.44E-60 |
| ICAM3 | pink | 0.850785 | 8.94E-51 | 0.88642 | 1.94E-60 |
| IL7R | pink | 0.870561 | 8.76E-56 | 0.885793 | 3.06E-60 |
| TMEM273 | pink | 0.847048 | 6.55E-50 | 0.885285 | 4.41E-60 |
| CORO1A | pink | 0.872633 | 2.35E-56 | 0.884876 | 5.91E-60 |
| DPEP2 | pink | 0.878771 | 4.12E-58 | 0.884865 | 5.95E-60 |
| NCF1 | pink | 0.884773 | 6.36E-60 | 0.884646 | 6.96E-60 |
| LAIR1 | pink | 0.887553 | 8.51E-61 | 0.884451 | 8.00E-60 |
| MS4A6A | pink | 0.867018 | 7.91E-55 | 0.884397 | 8.31E-60 |
| SAMSN1 | pink | 0.884347 | 8.61E-60 | 0.883486 | 1.59E-59 |
| P2RY10 | pink | 0.853565 | 1.96E-51 | 0.883435 | 1.64E-59 |
| LSP1 | pink | 0.898215 | 2.26E-64 | 0.883069 | 2.13E-59 |
| PRKCB | pink | 0.830563 | 2.36E-46 | 0.882029 | 4.40E-59 |
| CD247 | pink | 0.873976 | 9.87E-57 | 0.881062 | 8.61E-59 |
| CD226 | pink | 0.840456 | 1.94E-48 | 0.880087 | 1.68E-58 |
| TNFAIP8L2 | pink | 0.878687 | 4.36E-58 | 0.879843 | 1.99E-58 |
| CD1D | pink | 0.84002 | 2.41E-48 | 0.879507 | 2.50E-58 |
| PIK3CD | pink | 0.839208 | 3.62E-48 | 0.879181 | 3.12E-58 |
| CD38 | pink | 0.852985 | 2.70E-51 | 0.878613 | 4.58E-58 |
| CYTH4 | pink | 0.880927 | 9.45E-59 | 0.878081 | 6.56E-58 |
| MYO1G | pink | 0.866203 | 1.30E-54 | 0.877257 | 1.14E-57 |
| CD27 | pink | 0.850376 | 1.11E-50 | 0.876203 | 2.29E-57 |
| CCR4 | pink | 0.847674 | 4.71E-50 | 0.875832 | 2.93E-57 |
| GZMK | pink | 0.842081 | 8.53E-49 | 0.875418 | 3.85E-57 |
| IL21R | pink | 0.870397 | 9.71E-56 | 0.875287 | 4.19E-57 |
| SP140 | pink | 0.871124 | 6.14E-56 | 0.875231 | 4.35E-57 |
| LY9 | pink | 0.841624 | 1.08E-48 | 0.874809 | 5.74E-57 |
| SLC9A9 | pink | 0.80486 | 1.65E-41 | 0.874086 | 9.19E-57 |
| LAPTM5 | pink | 0.889587 | 1.89E-61 | 0.874014 | 9.63E-57 |
| CYTIP | pink | 0.897499 | 4.04E-64 | 0.873922 | 1.02E-56 |
| FAM49A | pink | 0.851619 | 5.69E-51 | 0.87195 | 3.63E-56 |
| CD3D | pink | 0.877331 | 1.08E-57 | 0.871381 | 5.22E-56 |
| SRGN | pink | 0.854929 | 9.20E-52 | 0.870938 | 6.90E-56 |
| HLA-DOA | pink | 0.850794 | 8.90E-51 | 0.870857 | 7.26E-56 |
| HLA-DMB | pink | 0.89498 | 3.01E-63 | 0.870591 | 8.59E-56 |
| CYBB | pink | 0.879124 | 3.24E-58 | 0.869894 | 1.33E-55 |
| CCR2 | pink | 0.84933 | 1.95E-50 | 0.869656 | 1.55E-55 |
| APBB1IP | pink | 0.8643 | 4.10E-54 | 0.867743 | 5.07E-55 |
| GPR171 | pink | 0.843427 | 4.29E-49 | 0.867594 | 5.56E-55 |
| CD84 | pink | 0.853918 | 1.61E-51 | 0.867203 | 7.07E-55 |
| LCP1 | pink | 0.856762 | 3.29E-52 | 0.866621 | 1.01E-54 |
| AOAH | pink | 0.845176 | 1.74E-49 | 0.864361 | 3.96E-54 |
| IRF4 | pink | 0.835152 | 2.65E-47 | 0.863046 | 8.67E-54 |
| DOK3 | pink | 0.850208 | 1.22E-50 | 0.862056 | 1.55E-53 |
| TFEC | pink | 0.861975 | 1.63E-53 | 0.861936 | 1.67E-53 |
| DOK2 | pink | 0.869719 | 1.49E-55 | 0.860848 | 3.16E-53 |
| HLA-DPB1 | pink | 0.863593 | 6.26E-54 | 0.858007 | 1.62E-52 |
| CD69 | pink | 0.84027 | 2.13E-48 | 0.857755 | 1.87E-52 |
| CD52 | pink | 0.875047 | 4.91E-57 | 0.857705 | 1.93E-52 |
| CD86 | pink | 0.867058 | 7.72E-55 | 0.856126 | 4.71E-52 |
| JAML | pink | 0.860285 | 4.38E-53 | 0.855891 | 5.37E-52 |
| CCR7 | pink | 0.806863 | 7.34E-42 | 0.855764 | 5.77E-52 |
| RIPOR2 | pink | 0.816899 | 1.10E-43 | 0.855168 | 8.05E-52 |
| CASS4 | pink | 0.818169 | 6.38E-44 | 0.854915 | 9.28E-52 |
| CD8A | pink | 0.822464 | 9.63E-45 | 0.853887 | 1.64E-51 |
| TRAT1 | pink | 0.830548 | 2.38E-46 | 0.853351 | 2.21E-51 |
| WIPF1 | pink | 0.851092 | 7.57E-51 | 0.850968 | 8.10E-51 |
| CXCR6 | pink | 0.840363 | 2.03E-48 | 0.850759 | 9.07E-51 |
| SELL | pink | 0.836377 | 1.46E-47 | 0.850499 | 1.04E-50 |
| CSF2RA | pink | 0.852631 | 3.27E-51 | 0.85028 | 1.17E-50 |
| SUSD3 | pink | 0.839053 | 3.91E-48 | 0.850207 | 1.22E-50 |
| CTLA4 | pink | 0.854464 | 1.19E-51 | 0.849713 | 1.59E-50 |
| BTLA | pink | 0.802888 | 3.62E-41 | 0.848435 | 3.15E-50 |
| GPR183 | pink | 0.843493 | 4.15E-49 | 0.846731 | 7.74E-50 |
| HLA-DRA | pink | 0.863516 | 6.55E-54 | 0.846664 | 8.02E-50 |
| CST7 | pink | 0.85238 | 3.76E-51 | 0.844413 | 2.59E-49 |
| LRRC25 | pink | 0.841395 | 1.21E-48 | 0.843596 | 3.94E-49 |
| FGR | pink | 0.839948 | 2.50E-48 | 0.843096 | 5.09E-49 |
| CCL5 | pink | 0.869654 | 1.55E-55 | 0.842997 | 5.35E-49 |
| HVCN1 | pink | 0.816536 | 1.29E-43 | 0.842149 | 8.24E-49 |
| LILRB2 | pink | 0.836421 | 1.43E-47 | 0.840464 | 1.93E-48 |
| IL2RB | pink | 0.838321 | 5.62E-48 | 0.840434 | 1.96E-48 |
| HCK | pink | 0.854706 | 1.04E-51 | 0.839603 | 2.97E-48 |
| FOXP3 | pink | 0.839031 | 3.95E-48 | 0.837633 | 7.89E-48 |
| NCF4 | pink | 0.851804 | 5.15E-51 | 0.837499 | 8.42E-48 |
| EOMES | pink | 0.815074 | 2.42E-43 | 0.83618 | 1.61E-47 |
| THEMIS | pink | 0.803559 | 2.77E-41 | 0.835037 | 2.80E-47 |
| LST1 | pink | 0.859267 | 7.88E-53 | 0.834395 | 3.82E-47 |
| AIF1 | pink | 0.846334 | 9.53E-50 | 0.833601 | 5.59E-47 |
| MNDA | pink | 0.834106 | 4.39E-47 | 0.833041 | 7.31E-47 |
| SIGLEC10 | pink | 0.804814 | 1.68E-41 | 0.832999 | 7.46E-47 |
| HLA-DQA1 | pink | 0.83779 | 7.30E-48 | 0.832237 | 1.07E-46 |
| SPI1 | pink | 0.838994 | 4.02E-48 | 0.828661 | 5.75E-46 |
| MS4A7 | pink | 0.800668 | 8.68E-41 | 0.826762 | 1.38E-45 |
| HLA-DPA1 | pink | 0.819032 | 4.38E-44 | 0.826616 | 1.48E-45 |
| NLRP3 | pink | 0.805216 | 1.43E-41 | 0.825939 | 2.01E-45 |
| CCR1 | pink | 0.815784 | 1.78E-43 | 0.825478 | 2.48E-45 |
| SLAMF6 | pink | 0.838983 | 4.04E-48 | 0.824249 | 4.32E-45 |
| CD6 | pink | 0.816819 | 1.14E-43 | 0.82409 | 4.65E-45 |
| MS4A4A | pink | 0.809837 | 2.17E-42 | 0.822316 | 1.03E-44 |
| HAVCR2 | pink | 0.843478 | 4.18E-49 | 0.821837 | 1.27E-44 |
| PDCD1 | pink | 0.806056 | 1.02E-41 | 0.818616 | 5.25E-44 |
| LILRB4 | pink | 0.829215 | 4.44E-46 | 0.816321 | 1.42E-43 |
| IKZF3 | pink | 0.803637 | 2.69E-41 | 0.816305 | 1.43E-43 |
| IGSF6 | pink | 0.839855 | 2.62E-48 | 0.814756 | 2.77E-43 |
| FYB1 | pink | 0.80551 | 1.27E-41 | 0.814744 | 2.78E-43 |
| C3AR1 | pink | 0.802029 | 5.08E-41 | 0.814236 | 3.45E-43 |
| CXCR4 | pink | 0.831491 | 1.53E-46 | 0.812495 | 7.19E-43 |
| FPR3 | pink | 0.810107 | 1.94E-42 | 0.81138 | 1.15E-42 |
| CIITA | pink | 0.815403 | 2.10E-43 | 0.808674 | 3.51E-42 |
| LY96 | pink | 0.831048 | 1.88E-46 | 0.80494 | 1.60E-41 |
| SLAMF8 | pink | 0.829462 | 3.96E-46 | 0.804692 | 1.76E-41 |
| NFAM1 | pink | 0.806595 | 8.19E-42 | 0.802063 | 5.01E-41 |
| FCGR2B | pink | 0.825333 | 2.65E-45 | 0.800379 | 9.72E-41 |
