# Supplementary material for: Gene coexpression network approach to develop an immune prognostic model for pancreatic adenocarcinoma
Source: World J Surg Oncol. 2021 Apr 12;19:112. doi: 10.1186/s12957-021-02201-w (PMC8042890; doi:10.1186/s12957-021-02201-w)
Supplement: Supplementary file 3 — Additional file 3: Table S2. Ten genes with both a log-rank P ≤ 0.2 and likelihood P ≤ 0.2 in the univariate analysis. [file 12957_2021_2201_MOESM3_ESM.docx]

**Table S2**

**Ten genes with both a log-rank *P* ≤ 0.2 and likelihood *P* ≤ 0.2 in the univariate analysis.**

| Object | HR | HR_CI_025 | HR_CI_975 | logrank_pvalue | Likelihood_pvalue |
| --- | --- | --- | --- | --- | --- |
| FCGR2B | 1.214125 | 1.010975 | 1.458096 | 0.037496 | 0.031747 |
| CD1D | 0.827765 | 0.65712 | 1.042724 | 0.108015 | 0.106458 |
| CCR7 | 0.8716 | 0.754452 | 1.006938 | 0.0618 | 0.056287 |
| ICAM3 | 0.837919 | 0.677271 | 1.036674 | 0.103384 | 0.097356 |
| HLA.DRA | 1.13986 | 0.956151 | 1.358866 | 0.143908 | 0.133591 |
| TESPA1 | 0.878238 | 0.72065 | 1.070285 | 0.19831 | 0.194566 |
| IL10RA | 0.8583 | 0.70133 | 1.050403 | 0.138157 | 0.139981 |
| HAVCR2 | 1.132919 | 0.933679 | 1.374675 | 0.205655 | 0.199334 |
| RASAL3 | 0.859349 | 0.697936 | 1.058093 | 0.154051 | 0.151426 |
| FAM78A | 0.842505 | 0.670841 | 1.058098 | 0.140486 | 0.137948 |
